# Effect of surgical approach for contralateral side hip arthroplasty in below knee amputees: a retrospective cohort study

**DOI:** 10.1186/s12891-018-2385-z

**Published:** 2019-01-05

**Authors:** Hong-Man Cho, Jae-Woong Seo, Hyun-Ju Lee, Sun-Do Kim, Kyu-Bok Kang, Jung-Ryul Kim, Ho-Wook Wee

**Affiliations:** 1 Department of Orthopedic Surgery, Gwangju Veterans Hospital, Gwangju, Cheomdanwolbong-ro 99, Gwangsan-gu, Gwangju, 62284 South Korea; 2 Department of Orthopedic Surgery, Veterans Health Service Medical Center, Seoul, Korea, South Korea; 3 Department of Orthopedic Surgery, Busan Veterans Hospital, Busan, South Korea; 4 Department of Orthopedic Surgery, Daegu Veterans Hospital, Daegu, South Korea

**Keywords:** Below knee amputation, Hip arthroplasty, Approach, Gluteus medius muscle

## Abstract

**Background:**

The gluteus medius muscle plays a very important role in the stability of the gait, especially in patients with amputation of the lower limbs. Therefore, choosing the appropriate type of approach for hip arthroplasty is very important. Hence, this study aimed to compare the outcomes and complications between the anterolateral approach (ALA) and posterior approach (PA) for hip arthroplasty in patients with contralateral below knee amputation.

**Methods:**

From January 1999 to November 2014, 67 patients who underwent hip arthroplasty with contralateral below knee amputation were retrospectively analyzed. The study subjects were divided into two groups: the PA group (33 cases) and the ALA group (34 cases). The results of the clinical functional recovery with Western Ontario and McMaster Universities Osteoarthritis Index (WOMAC) score, Harris Hip Score, and activity of daily living scale were compared between the two groups. During the follow-up period, complications related to gait such as fall, dislocation, and periprosthetic fractures (PPFs) were investigated.

**Results:**

The Harris Hip Score (*p* = 0.024) and the activity of the daily living scale (*p* = 0.043) of the ALA group were significantly lower at 3 months compared to the PA group, but no significant difference was observed between the two groups from 6 months postoperatively to the last follow-up. The WOMAC score was not significantly different between the two groups. Within 3 months after surgery, falls occurred in 3 cases in the PA group and in 11 cases in the ALA group (*p* = 0.019) Dislocation and PPF were caused by prosthesis-related trauma. Two dislocations and 1 PPF occurred 8 years postoperatively in the PA group. PPF occurred in 3 patients in the ALA group, of which 2 occurred within 3 months after surgery.

**Conclusion:**

Orthopedic surgeons should pay particular attention in patients with hip arthroplasty on the contralateral side hip who had below knee amputation because functional recovery is delayed until 3 months after ALA compared with PA.

## Background

Surgical operations for lower limb amputation are performed in cases where necrosis of the lower limb (caused by tumors, congenital abnormalities, and vascular diseases) is observed or in cases where salvage surgery is contraindicated owing to severe trauma or infection. Most amputees are successful ambulators; hence, most are exposed to long-term prosthetic use [1]. For ambulation of amputees using prosthesis, muscle power of the contralateral side hip joint flexor muscle and abductor muscles is more important than the power needed for the ambulation of non-amputees [2].

The surgical approach for hip arthroplasty (HA) remains a controversial topic. Several surgical approaches are available that can be used to perform HA. The most commonly used approaches are the posterior approach (PA) and anterolateral approach (ALA), each with distinct advantages and disadvantages. The PA is considered easier to perform and is generally a quicker procedure, limiting operative complications such as blood loss and anesthetic issues. The abductor muscles are not disturbed significantly, so there is generally no gait abnormality [3]. Nevertheless, PA has several limitations. The main drawback is the damage to the short external rotators of the hip, which increases the risk of postoperative instability [4]. Acetabular exposure is limited, and it is known that there is a risk of sciatic nerve damage [5] especially when extensive hip revisionary surgery is performed with PA [6].

An advantage of ALA is a decreased incidence of dislocations. However, the ALA has some drawbacks. During the approach, the anterior part of the gluteus medius muscle makes it difficult to visualize the acetabulum and femur sufficiently. Therefore, tenotomy is essential [7] and it is possible to attenuate abduction strength after surgery. Moreover, in this approach, there is a risk of injury to the inferior branch of the superior gluteal nerve [8]. If this nerve is damaged, abduction weakening becomes more severe [9], and the patient may be forced to limp walking after surgery [10]. Therefore, this may severely decrease the patient’s satisfaction with the procedure [11]. Efforts have been made to minimize damage to the gluteus medius muscle. Bertin [10] and Higuchi et al. [12] introduced a minimally invasive ALA that approaches the plane between the gluteus medius muscle and tensor fascia lata that minimizes gluteus medius damage. Minimally invasive ALA provides rapid rehabilitation after surgery and also has the advantage of being able to prevent posterior dislocation due to the lack of damage to the posterior articular capsule. However, it is not widely used because it has a steep learning curve as well as problems due to the high soft tissue tension during the procedure. Fortunately, muscle weakness due to damage of the gluteus medius muscle from using ALA is known to be temporary. Winter et al. [13] performed total hip arthroplasty using PA, ALA, and the anterior approach and reported measurements of muscle strength. The leg press power and abduction strength were significantly lower 6 weeks postoperatively in cases using ALA rather than PA, but there was no difference after 3 months. The function of the gluteus medius is more important when considering the gait characteristics of the limb amputee, which support mainly the single limb. Therefore, when performing HA in the contralateral hip of the amputated lower limb, it is important to consider the injury of the gluteus medius muscle, which is known as the powerful abductor muscle in the surgical approach. This is because, as was observed in Winter’s study, if muscle strength is reduced for a certain period after surgery, it is likely that a lower limb amputee at high-risk of falling is likely to experience a fall that can have a significant impact on postoperative quality of life during this period. Herein, we present a retrospective series of HAs after contralateral lower extremity amputation. Although the authors’ study did not directly measure muscle strength postoperatively in each period, it is believed that each approach (PA and ALA) possibly affects the postoperative hip joint function, recovery of gait ability, and occurrence of accidents such as falls.

## Methods

The study included patients with leg amputations who underwent total hip arthroplasty for the contralateral hip joint from January 1999 to November 2014, under four hip arthroplasty specialists affiliated to four different hospitals. The patients who had undergone HA less than 5 years previously and patients who could not walk independently and were unable to perform all social activities prior to surgery were excluded. Operations via the PA were performed with the patient in the lateral decubitus position. The short external rotator muscles, including the piriformis tendon and the capsule, were resected with a single flap. The joint was reduced after the cups and stems were inserted. The range of motion, torsion, and stability of the soft tissues were examined, and the tissue flap containing the articular capsule was directly sutured to the posterior part of the proximal femur. In this study, all ALAs were performed with the patient under the lateral decubitus position. After the subfascial space is entered, the gluteus maximus is split via blunt dissection, and a smaller dissection of only one-half to two-thirds of the gluteus medius and gluteus minimus from the anterior border of their insertion to the greater trochanter is created. The hospitals where the authors perform surgery using a computerized common medical record system and apply the same postoperative management manuals for the same operations. The patient data were collected and analyzed retrospectively using the computerized medical records of each hospital.

### Perioperative evaluation

Perioperative blood loss volume (intraoperative blood loss volume was calculated by subtracting the normal saline volume used for irrigation at the total fluids volume contained in suction drain bottles and adding weight gain of gauzes used at the time of surgery; intraoperative blood loss volume was calculated by adding postoperative drain volume), operation time, and the postoperative blood transfusion volume were examined. The total hospital periods from admission to discharge were compared.

### Functional evaluation

After the surgery, the timing of the start of ambulation using walking aids such as walkers or crutches was evaluated. The results of functional recovery were evaluated immediately after surgery, 3 months, 6 months, 1 year after surgery, and then annually afterward. The Harris Hip Score (HHS) [14] for pain, ambulation, and degrees of movements and the Western Ontario and McMaster Universities Osteoarthritis Index (WOMAC) [15] were used for the evaluation. Postoperative activities of daily living (ADL) scale [16] were used for functional recovery check.

### Complications

To compare early stage ambulation stability after surgery, the patients who experienced a fall from surgery to 1 year were evaluated. A fall is defined as “an event which resulted in the person coming to rest inadvertently on the ground or other level, other than as a consequence of lost consciousness, a violent blow, stroke or epileptic seizure” [17]. Moreover, infections, dislocation, periprosthetic fractures (PPFs), implants loosening, and revision surgeries that may or may not affect ambulation were evaluated. Also, we compared the results of hip arthroplasty in the amputee group with the nonamputee group with regards to the incidence of falls, dislocation, PPFs, and loosening. The incidence of complications between the two groups was compared and analyzed by matching them based on variables such as the surgeon, duration of the operation, age, approach, and inserted implants.

### Statistical analysis

Statistical analyses were conducted with IBM-SPSS 18.0 software (IBM-SPSS, Armonk, NY, USA). For comparison between groups, we used a non-parametric Mann–Whitney U-test for continuous variables (such as preoperative evaluation and functional evaluation) and the chi-square test for categorical variables (such as fall, dislocation, and PPFs). Kaplan-Meier survival analyses were conducted using dislocation, periprosthetic fractures, and implant loosening as the end point for comparisons within each group. Statistical significance was set at *p* < 0.05.

## Ethics statement

The design and protocol of this retrospective study were approved by the institutional review board of the author’s hospital (GJVH-IRB No. 2016-11-5). All patients were informed that their medical data could be used in a scientific study and provided their consent.

## Results

### Case analysis and perioperative evaluation

From January 1999 to November 2014, 74 HAs were performed for 74 patients (72 men and 2 women) on the contralateral hip and lower limb below-knee amputation at the author’s institutions. Seven patients were excluded because they were not followed up for less than 5 years after surgery. In one case, the patient died of lung cancer 18 months after the surgery. The other case was excluded from the study because the patient was admitted to a dementia care facility within 4 months after the surgery and was unable to perform any social activities. The remaining 67 patients (all men, as the hospital treats soldiers injured during training or combat), met the inclusion criteria. The surgery was performed by four orthopedic surgeons who majored speciality in hip arthroplasty. All patients in both group were treated with third generation cephalosporin for 3 days after surgery. For all patients, except those at risk of endogenous hemorrhage, low molecular weight heparin was administered for preventing deep vein thrombosis from the first postoperative day until discharge at 2 weeks after surgery. The average follow-up period was 84.3 months (minimum, 60 months; maximum, 180 months). The PA was used in 33 cases, and the ALA was used in 34 cases (Table 1). Among the 67 patients, 57 and 10 patients underwent total hip arthroplasty and bipolar hemiarthroplasty, respectively. The preoperative diagnoses before HA in 32, 26, and 9 patients were hip joint osteoarthritis, avascular necrosis of the femoral head, and femur neck fracture, respectively. The average age of the patients at the time of surgery was 69.7 years (range, 54–82 years), and the average body mass index (BMI) was 26.2 kg/m^2^ (range, 18.6–35.1 kg/m^2^). Cementless cups and stems were implanted in all hips. Among the patients who underwent total hip arthroplasty, a ceramic-on-ceramic bearing was used in 44 hips (77.2%), and a metal-on-polyethylene bearing was used in 13 hips (22.8%). The mean surgical time was 82.5 min (45–115 min) in the PA group and 83.2 min (45–125 min) in the ALA group. No significant difference was observed in the operation time (*p* = 0.871). No significant difference was observed (*p* = 0.798) in perioperative blood loss between the groups [923.6 ml (330–2030 ml) in the PA group and 919.7 ml (380–2150 ml) in the ALA group]. In addition, no significant difference was observed in the amount of blood transfusion from 1.14 units (0–3) in the PA group to 1.02 units (0–3) in the ALA group (*p* = 0.878). The average hospital period is 17.1 days (8–24) in the PA group and 18.5 days (7–25) in the ALA group (Table 2).

**Table 1:** Characteristics of the included patients

		PA group	ALA group	*p* value
Number of cases		33	34	
Sex		All male	All male	
Age (years)		69.4 (54–80)	69.9 (55–82)	0.976
BMI (kg/m^2^)		26.3 (19.1–35.1)	26.2 (18.6–34.7)	0.879
Diagnosis	Primary osteoarthritis	15	17	
	Avascular necrosis	14	12	0.841
	Femur neck fx	4	5	
Operation	Total hip arthroplasty	28	29	0.959
	Bipolar hemiarthroplasty	5	5	
Bearing	Ceramic-ceramic	20	24	0.308
	Polyethylene-metal	8	5	
Follow-up (months)		84.0 (60–180)	84.5 (60180)	0.617

*PA* posterolateral approach, *ALA* anterolateral approach, *BMI* body mass index, *Fx* fracture

**Table 2:** Clinical results and complications of the PA and ALA groups

		PA group	ALA group	*p* value
Number of cases		33	34	
Perioperative evaluation	Operation time (minutes)	82.5 (45–115)	83.2 (45–125)	0.871
	Bleeding (ml)	923.6 (330–2030)	919.7 (380–2150)	0.798
	Transfusion (units)	1.14 (0–3)	1.02 (0–3)	0.878
	Hospital days	17.1 (8–24)	18.5 (7–25)	0.765
Functional evaluation	Ambulation (start day)	5.79 (3–12)	5.51 (3–10)	0.789
	Harris hip score			
	3 months	80.83 (70–96)	74.51 (64–92)	0.024
	6 months	89.55 (76–100)	88.72 (74–97)	0.544
	12 months	93.62 (84–100)	91.3 (86–97)	0.852
	ADL scale			
	3 months	3.88 (3–5)	2.45 (2–5)	0.043
	6 months	4.76 (4–6)	4.95 (4–6)	0.575
	12 months	5.86 (4–6)	5.67 (4–6)	0.891
	WOMAC scale			
	3 months	19.86 ± 3.52	21.26 ± 4.86	0.599
	6 months	25.13 ± 2.15	26.85 ± 1.63	0.626
	12 months	27.34 ± 5.27	29.49 ± 7.25	0.973
	Fall (within 3 months)	3	11	0.019
Complications	Dislocation	2	0	0.145
	Periprosthetic Fx	1	3	0.317
	within 3 months	0	2	0.157

*PA* posterolateral approach, *ALA* anterolateral approach, *ADL* activity of daily living, *WOMAC* Western Ontario and McMaster Universities Osteoarthritis Index, *Fx* fracture

### Functional evaluation

**Fig. 1:**
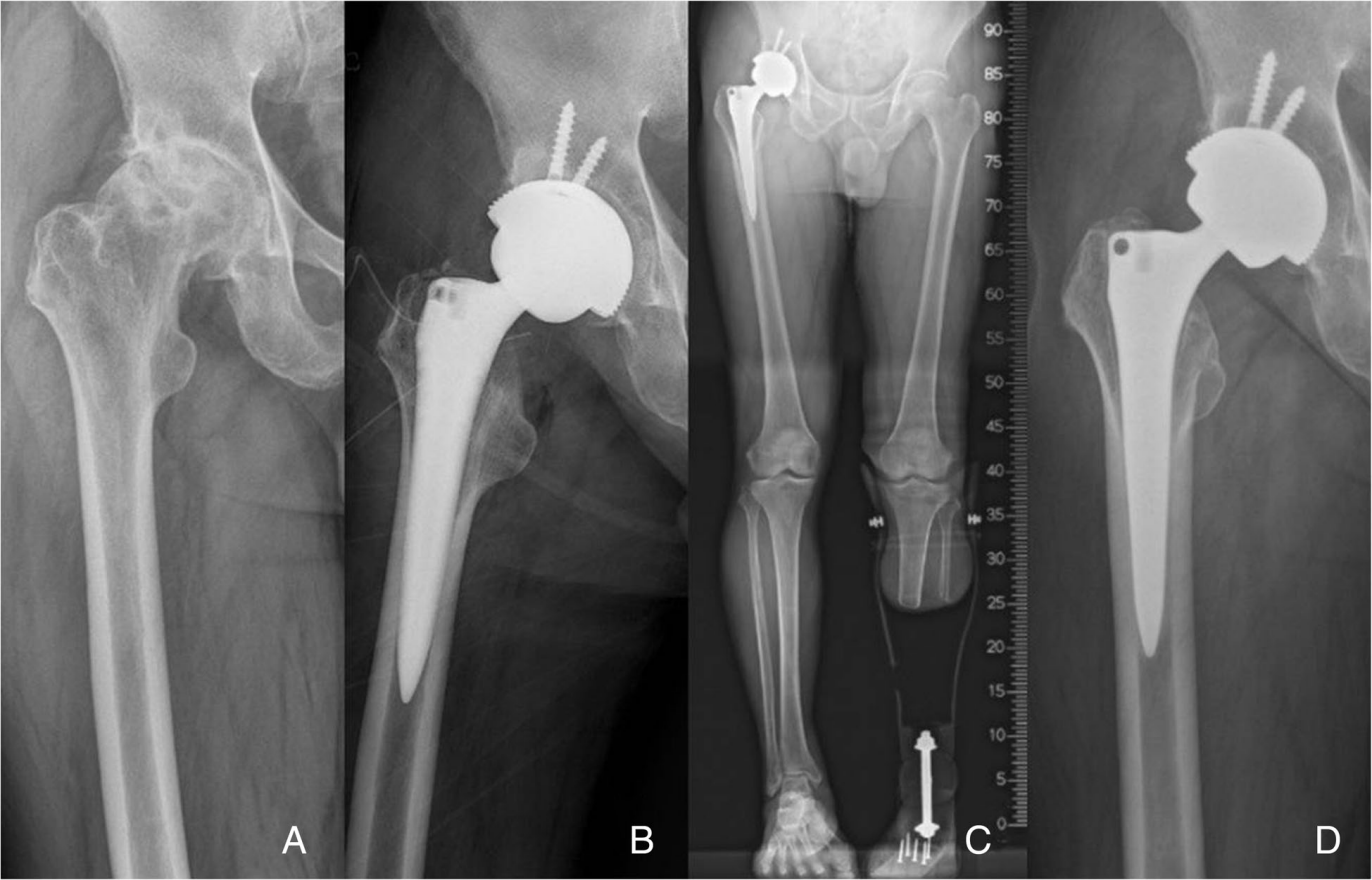
**a** Preoperative radiograph of a 73-old-year man with primary osteoarthritis of the hip joint. **b** Postoperative radiograph shows the excellent implant position of cementless total hip arthroplasty using the posterolateral approach. **c** Postoperative whole lower extremity radiograph of the prosthetic leg taken while standing. **d** At 5 years after the operation, the radiograph shows stable fixation of the components with a radiolucent line around the proximal femoral stem

**Fig. 2:**
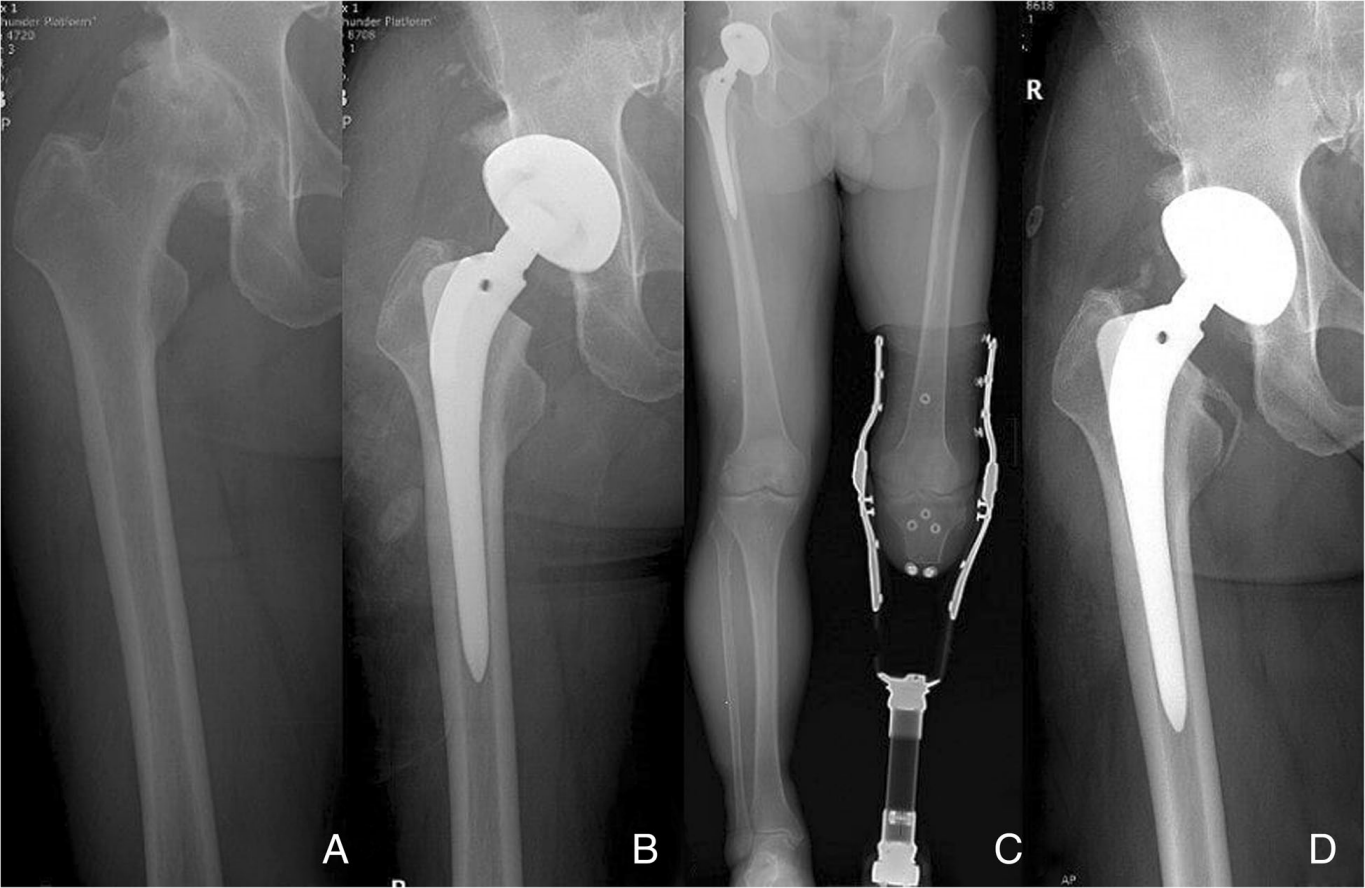
**a** Preoperative radiograph of a 67-old-year man with primary osteoarthritis of the hip joint. **b** Postoperative radiograph shows the excellent implant position of cementless total hip arthroplasty using the anterolateral approach. **c** Postoperative whole lower extremity radiograph of the prosthetic leg taken while standing. **d** At 7 years after the operation, the radiograph shows stable fixation of components without subsidence or changes in alignment

No significant difference was observed in the mean time to start of ambulation using walking aids between the two groups. Clinically, the differences in the HHS [14] (*p* = 0.024) and ADL scale [16] (*p* = 0.043) between the two groups were significant at 3 months after surgery. However, no significant difference was found in the HHS [14] or in the ADL scale [16] between the PA group and the ALA at 6 months after operation to the final follow-up. The mean WOMAC score [15] was not significantly different between the two groups after surgery to the last follow-up (Table 2) (Figs. 1 and 2).

### Complications

#### Fall

Within 3 months after surgery. In the amputee group, falls occurred in 3 cases in the PA group and in 11 cases in the ALA group (*p* = 0.019). In the PA group, 2 falls occurred indoors and 1 occurred outdoors. Of the 11 cases in the ALA group, 9 falls occurred indoors and 2 occurred outdoors. Three cases of falls occurred during walking activity outdoors (PA group, 1; ALA group, 2 cases), and 11 cases that were related to prosthesis occurred indoors (PA group, 2 cases; ALA group, 9 cases).

#### Dislocation

In the amputee group, there were 2 cases of dislocation in the PA group. All 2 cases occurred in patients who underwent total hip arthroplasty. Dislocation occurred at 4 and 8 weeks after surgery, respectively. One patient with a prosthesis on the amputated stump experienced dislocation while taking off the prosthesis while sitting on the floor, and the other one with a prosthesis slipped on the doorstep during ambulation. Both patients underwent closed reduction, and no redislocation occurred.

#### Periprosthetic fracture

**Fig. 3:**
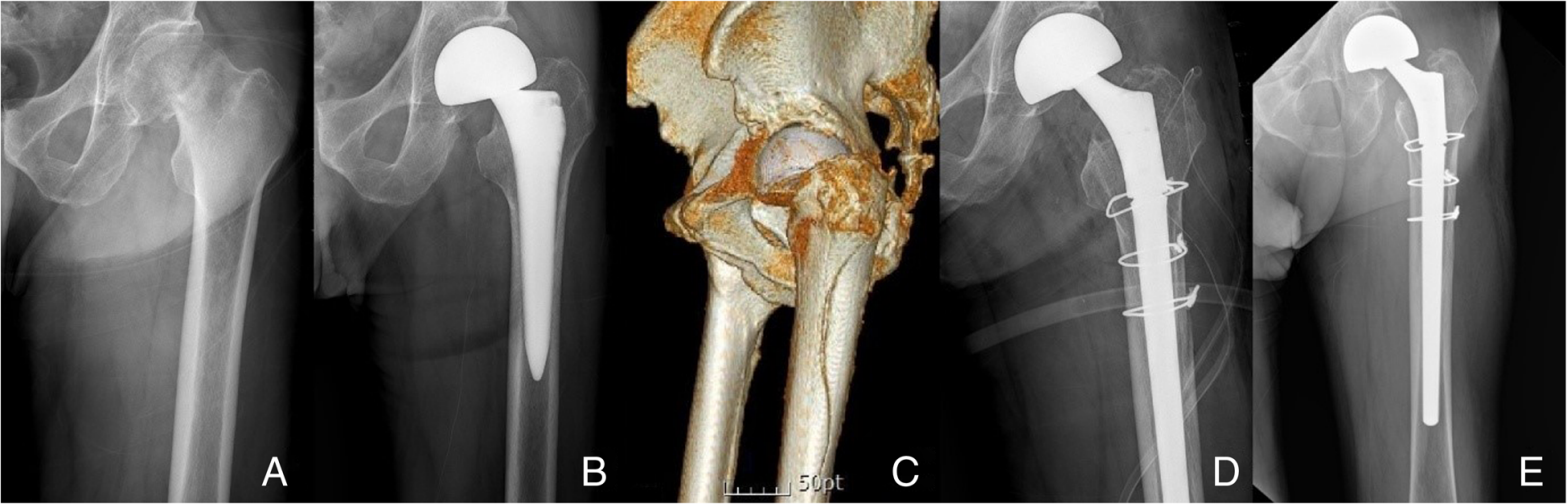
**a** Preoperative radiograph of a 70-old-year man with subcapital femoral neck fractures. **b** Postoperative radiograph shows the excellent implant position of cementless bipolar hip arthroplasty. **c** A Vancouver type B fracture with stem loosening occurred after a fall at 2 months postoperatively. **d** Stem revision was performed using a long distal-fitting and modular-type stem with additional circular wiring. **e** At 24 months after revision surgery, the radiograph showed bony union and stable stem fixation

In amputee group, PPF around the femoral stem occurred in 1 patient in the PA group and in 3 patients in the ALA group. All 4 patients developed prosthesis-related trauma. Two of the 3 patients in the ALA group experienced prosthesis-related trauma within 3 months after surgery (one patient developed a fracture when his pants were caught on his prosthesis while changing, whereas the other had a fracture when the patient suddenly stood up after wearing the prosthesis). Meanwhile, the remaining patient in the ALA group had a fracture 4 years following surgery after falling while walking on a slippery surface. One patient in the PA group 8 years after surgery had PPF after the prosthesis loosened during stair climbing. The site of PPF in 4 cases was around the femoral stem (Vancouver type B fracture) [18]. All 4 patients underwent revision surgery (Fig. 3).

#### Loosening

**Fig. 4:**
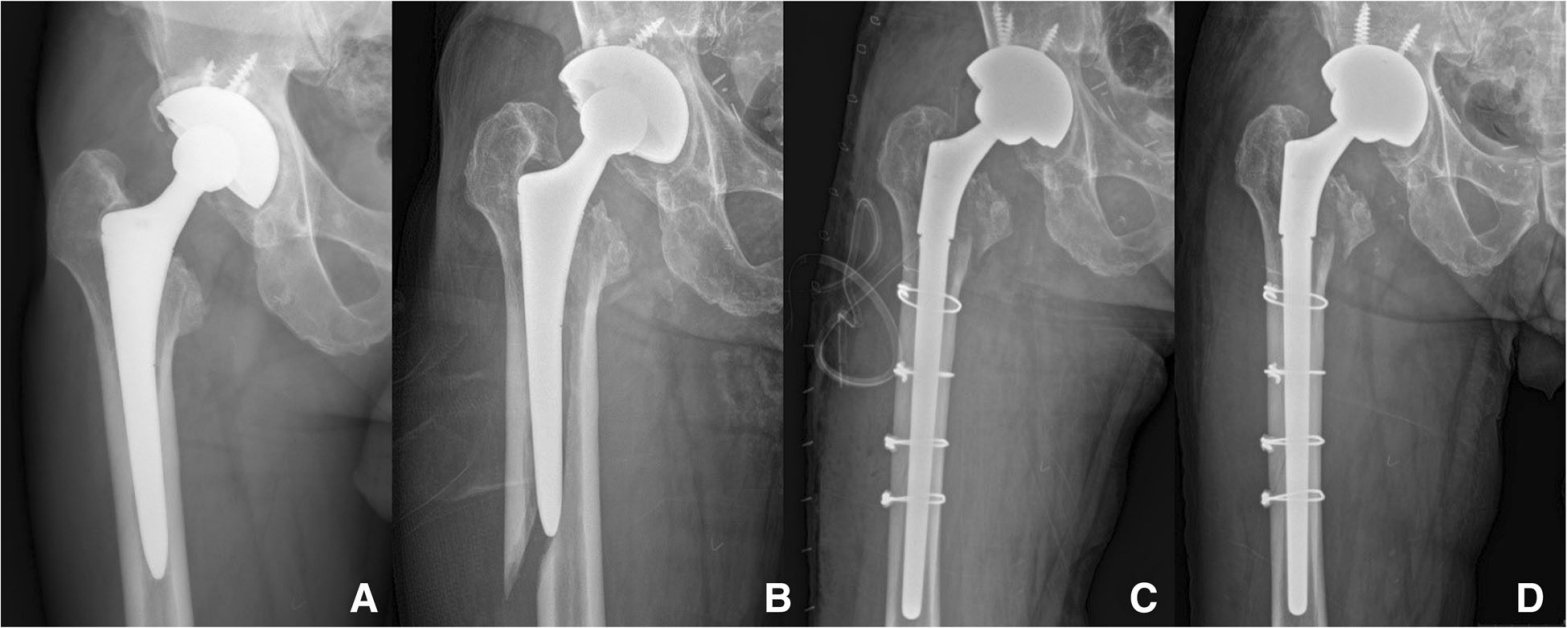
**a** Radiograph of the right hip joint 7 years after operation in a 71-old-year man who underwent left below knee amputation with total hip arthroplasty. The radiograph shows loosening of the components around the acetabulum and proximal femur. **b** A Vancouver type B2 fracture with stem loosening occurred after a fall at 8 years postoperatively. **c** Acetabular cup and femoral stem revision was performed using a long distal-fitting and modular-type stem with additional circular wiring. **d** At 24 months after revision surgery, the radiograph showed bony union and stable stem fixation

In amputee group, loosening of artificial joints (cup and stem) was confirmed in 1 patient in the PA group at 6 years after the operation. Revision surgery was recommended for loosening of the femoral stem and acetabular cup; however, the patient refused revision surgery and received conservative treatment without surgery. After 2 years after diagnosed loosening, PPF developed, and acetabular cup and femoral stem exchange was performed (Fig. 4).

#### Survival analysis of the complications between amputee group and non-amputee group

**Fig. 5:**
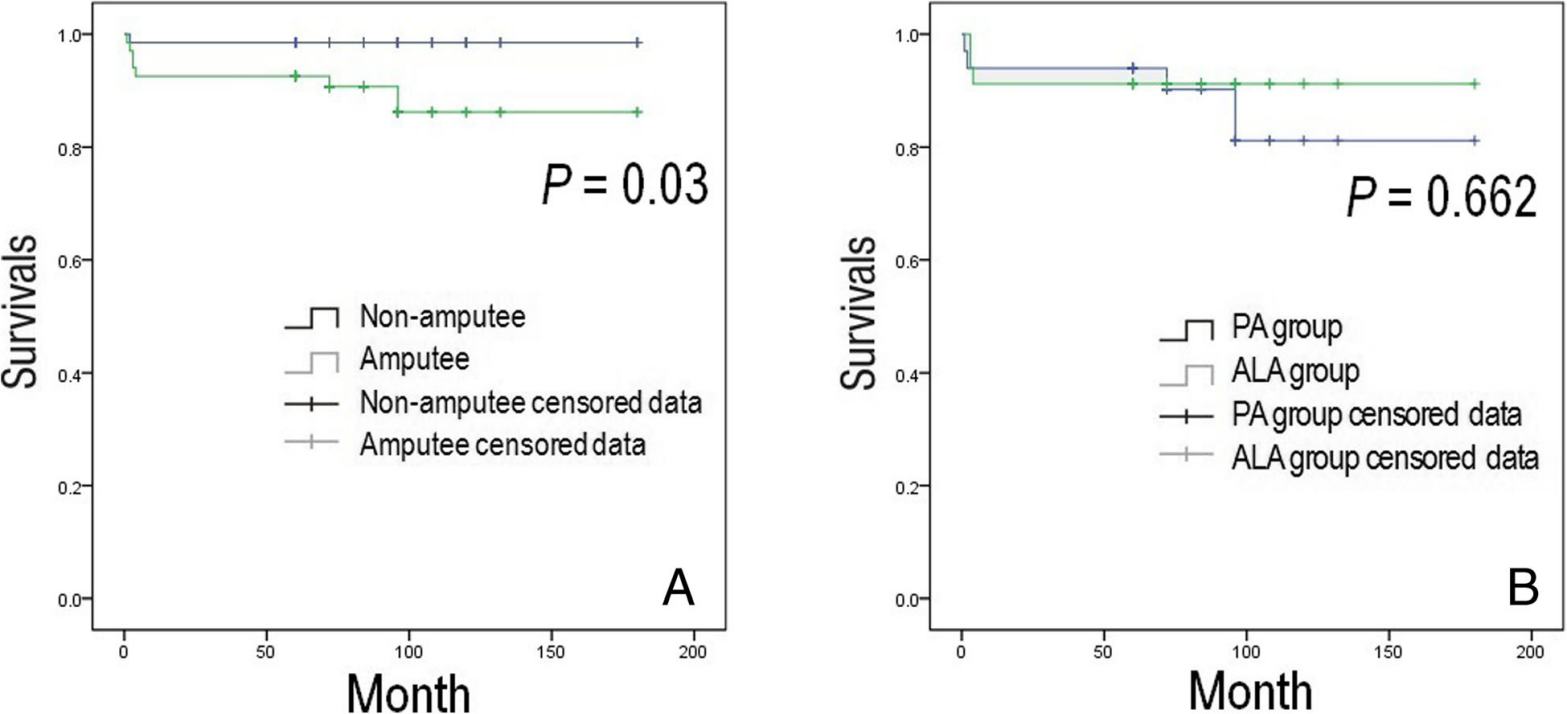
**a** Survival analysis of the complications between the amputee and non-amputee groups. Dislocation, PPF, and loosening were determined as the end point. Results of analysis shows a significant difference between the amputee and non-amputee groups (*p* = 0.030). **b** Survival analysis of the complications between the PA and ALA groups in the amputee group. Dislocation, PPF, and loosening were determined as the end point. There was no significant difference between the PA and ALA groups in the amputee group

Falls that occurred within 3 months after surgery were reported for 2 non-amputees (1 PA and 1 ALA) and 14 amputees (3 PA and 11 ALA), thus being significantly higher in the amputee group (*p* = 0.001). Dislocation occurred in 1 non-amputee (PA) and 2 amputees (both PA) and was not significantly different. PPF and implants loosening did not occur in the non-amputee group, but 4 PPFs (1 PA and 3 ALA) and 1 loosening (PA) occurred in the amputee group. PPF occurred significantly more frequently in the amputee group (*p* = 0.042), while loosening was not significantly different between the two groups (*p* = 0.315) (Table 3). Dislocation, PPF, and loosening were determined as endpoints. In the survival analysis, there was no significant difference between PA and ALA in the amputee group, but there was a significant difference between the amputee and non-amputee groups (*p* = 0.030) (Fig. 5).

**Table 3:** Complications of the Amputee and the Non-amputee groups

		Amputee group	Non-amputee group	*p* value
Fall (< 3 months)	Total	14	2	0.001
	PA group	3	1	
	ALA group	11	1	
Dislocation	Total	2	1	0.559
	PA group	2	1	
	ALA group	0	0	
Periprosthetic Fx	Total	4	0	0.042
	PA group	1	0	
	ALA group	3	0	
Loosening	Total	1	0	0.315
	PA group	1	0	
	ALA group	0	0	

*PA* posterolateral approach, *ALA* anterolateral approach, *Fx* fracture

#### Others

There were no postoperative infections in both groups, and none of the BHA patients showed acetabular erosion (Table 2).

## Discussion

There is a high risk of arthritis in the contralateral hip joint in patients with below knee amputations [19–21]. Kulkarni [20] et al. analyzed 44 cases of below knee amputations and reported that 18% of patients developed arthritis in the contralateral hip joint; this was double the incidence observed in non-amputated patients. Struyf et al. reported that hip arthritis on the contralateral side of the amputated leg was five- to ten-times higher and the progression of the arthritis was faster in patients with amputations than in those without [21]. The most common cause of arthritis in the contralateral hip joint in patients with below knee amputation was a change in their gait pattern owing to special circumstances, such as leg amputation and use of prosthetics (increased metabolic energy expenditure, decreased walking speed, larger stride width, shorter stride length with the intact limb, and increased stance time). It is known to induce pain and degenerative changes by inducing a higher load on the joint owing to an increase in the ground reaction forces. [2, 20, 22–24] The gluteus medius muscle is an important abductor muscle [25] that plays important roles in stabilizing the pelvis during the period of single support of the gait cycle [22], and in the balance and normal movement of the pelvis and lower limb during gait [26]. The role of the muscle abduction in the hip contralateral to the amputated leg becomes particularly more important for stable gait in patients with leg amputations. In this case, the anterior approach was used and the anterior part of the gluteus medius—which plays a major role in abduction, internal rotation, and flexion of the hip joint—was cut off [27]. Patients who underwent the anterior approach showed more falls within the postoperative 3 months, and slower functional recovery compared to those who underwent the posterior approach. Therefore, it was thought that damage to the anterior part of the gluteus medius by the anterolateral approach may have affected the outcomes.

The incidence of periprosthetic femoral fractures after hip arthroplasty is increasing [28]. A recent study showed that the incidence of periprosthetic femoral fracture is approximately 1% after primary HA [29]. The authors observed a higher incidence of periprosthetic femoral fractures in both groups (3% in the PA group (1 in 33 patients) and 8.8% in the ALA group (3 in 34 patients)). It is a well-known fact that a patient with amputation belongs to the high-risk group for fall [30]. Falls occurred more frequently in the ALA group (2 out of 3 patients) than in the PA group within 3 months after the operation. We believe that the delayed functional recovery of the ALA group (until 3 months postoperative) seems to be related to these results and the damage of the anterior fiber of the gluteus medius during ALA.

In a study of functional recovery based on the approach type, Jeya et al. [31], in a medium term (5 years), found no difference in the clinical benefit of surgery as defined by the change in Oxford Hip Score or in the absolute postoperative Oxford Hip Score between patients who underwent PA and with those who underwent ALA. However, the initial difference at 1 year in Oxford Hip Score between the PA and ALA groups may be attributed to the increased trochanteric pain [32] and increased gait abnormalities [33] in the ALA group during the immediate postoperative period. In particular, Pfirrmann et al. [32] found changes in the abductor muscle after hip arthroplasty using MRI. In the case of HA with partial incision of the gluteus medius and gluteus minimus, defects in the gluteus minimus and gluteus medius were observed in 8 and 16% of patients without postoperative trochanteric pain or limp symptoms, respectively; however, gluteus medius and gluteus minimus defects were found in 62% and in 56% of patients with symptoms such as trochanteric pain or limp, respectively. In the present study, functional recovery was lower in the ALA group than in the PA group until 3 months postoperatively, but no significant difference was observed between the two approaches from 6 months to the last follow-up. Therefore, we concluded that when HA is performed to the contralateral side hip joint of amputees, minimizing the gluteus medius damage and doing the best to repair it was necessary when using the ALA.

Amputees are a high-risk group for fall. Kulkarni et al. found that 60% of amputees reported that falling affected their daily life, work, leisure, and confidence [30]. These falls are due to altered lower limb mechanics; therefore, transtibial amputees make compensatory gait adjustments. In the present study, gluteus medius muscle damage in patients with high-risk falls could be an important risk factor for falls and PPF in the ALA group within 3 months after surgery.

Other notable findings are those of four patients with PPF (around the femoral stem) and of two patients with dislocation due to prosthetic leg-related falls, despite the absence of osteoporosis or problems with walking ability. Therefore, providing thorough education to patients to wear and use the prosthesis with caution after surgery is important. Since ALA and PA both have advantages and disadvantages, we do not believe that only one approach should be used exclusively for hip arthroplasty in the contralateral hip joint of below the knee amputees. However, the following points should be considered before surgery. First, it is important to use the approach that is the surgeon is most familiar with. Surgeons using ALA should minimize the damage of the gluteus medius muscle and the muscles around the hip joint and should operate quickly and safely. Surgeons using PA should reduce damage and do their best to repair structures that could affect the stability of the hip joint, such as the short external rotator. Second, as our results have shown, the risk of falls should be adequately explained to patients as there is a high risk of falls and fractures around the femoral stem in amputees compared with non-amputees. In particular, patients with ALA should be more careful because if they fall within the first 3 months after surgery they will have a slow recovery of gait ability. Last, patients with a high risk of falls (those living on the floor, those not expected to be well coordinated with postoperative care, those who need to return to active work soon after surgery, etc.) should have PA performed by a skilled surgeon.

This study has some limitations. First, the sample size is small. However, collecting data from many cases is difficult because hip arthroplasty of the contralateral side hip joint in patients with below knee amputation is rare. Second, the surgery was performed by 4 surgeons all of whom have > 10 years of experience with total hip arthroplasty and have performed > 300 surgeries per year. Third, the difference in muscle strength due to the injury to the gluteus medius, which was the most significant difference between the two approaches, was not identified. It was evaluated by comparing only events of falling or functional recovery. The study was further limited by its retrospective nature and the relatively small number of patients. Therefore, the results need to be supplemented by large-scale prospective studies. The final limitation is that the study included both patients with total hip replacement and with hemiarthroplasty. In particular, 9 out of 10 hemiarthroplasty patients had hemiarthroplasty due to femoral neck fracture. Most patients with femoral neck fracture were elderly and likely to have osteoporosis and often had reduced gait ability before fracture. Yet, because we selected patients who were socially active with prosthetics before the fracture, they were included in the study. It is, however, considered that a limitation of this study was the failure to distinguish the presumed complications that are more likely to occur in total replacement, such as dislocation.

## Conclusions

We believe that the approach should be chosen very carefully when performing arthroplasty in the contralateral hip joint of a below the knee amputee. In ALA surgery, functional recovery is delayed for at least 3 months after the procedure compared with PA and the risk of falls is higher. More attention, therefore, should be paid to postoperative patient care and education. It is thought that it is better to use PA in contralateral hip joint arthroplasty in patients with below the knee joint amputation due to the higher risk of falls.

## Abbreviations

ADL: Activities of daily living
ALA: Anterolateral approach
BMI: Body mass index
HA: Hip arthroplasty
HHS: Harris Hip Score
PA: Posterior approach
PPF: Periprosthetic fractures
WOMAC: Western Ontario and McMaster Universities Osteoarthritis Index
